# Pathophysiological role of endothelial biomarkers in *Bothrops* sp. venom-induced renal dysfunction and the therapeutic effect of antivenom

**DOI:** 10.1016/j.toxcx.2025.100226

**Published:** 2025-05-20

**Authors:** Nicole Coelho Lopes, Gdayllon Cavalcante Meneses, Ranieri Sales de Souza Santos, Leticia Machado de Araújo, Bruna Viana Barroso Martins, Katarina Maria dos Reis Araújo, Valéria Holanda Nogueira de Aquino, Igor Moreira de Almeida, Sandra Mara Brasileiro Mota, Geraldo Bezerra da Silva Junior, Camila Eleuterio Rodrigues, Elizabeth De Francesco Daher, Polianna Lemos Moura Moreira Albuquerque, Alice Maria Costa Martins

**Affiliations:** a Pharmacology Post-Graduate Program, Federal University of Ceará, Fortaleza, Ceará, Brazil; b Department of Clinical and Toxicological Analysis Federal University of Fortaleza, Fortaleza, Ceará, Brazil; c Medical Sciences Post-Graduate Program, Federal University of Ceará, Ceará, Brazil; d Toxicological Information and Assistance Center, Instituto Doutor Jose Frota Hospital, Fortaleza, Ceará, Brazil; e University of Fortaleza, School of Medicine, Fortaleza, Ceará, Brazil; f Hospital das Clínicas, University of São Paulo School of Medicine, São Paulo, Brazil; g University of New South Wales, Sydney, Australia; h Charles Darwin University, Darwin, Australia

**Keywords:** Snakebite, Antivenom, Kidney injury, Vascular cell adhesion molecule-1, Angiopoietins, KIM-1

## Abstract

Snakebite antivenom (SAV) is the standard treatment option to neutralize the toxic effects of snake venom, but their consequences on kidney function need to be better understood. This study aims to evaluate the effects of antivenom on kidney and endothelial biomarkers due to *Bothrops* venom in two subgroups of patients distinguished by the presence of hemorrhagic syndrome at admission. This prospective study included 34 snakebite patients admitted to a tertiary hospital in Northeast Brazil between August 2019 and November 2020, 50 % of whom experienced spontaneous bleeding. Endothelial and kidney damage biomarkers were analyzed at three time points: before antivenom infusion and after 10 h and 20 h of antivenom infusion. Bleeding patients exhibited higher urine Neutrophil Gelatinase-Associated Lipocalin (uNGAL) and Kidney Injury Molecule-1 (KIM-1) levels, indicating incomplete renal recovery until 20h after antivenom. This group showed higher serum angiopoietin-2 (Ang-2) levels and vascular cell adhesion molecule-1 (VCAM-1). VCAM-1 levels positively correlated with kidney biomarker levels at each time point, especially after SAV. uNGAL was variant across VCAM-1, Ang-1, and Ang-2 levels before antivenom. Elevated levels of uNGAL and KIM-1, observed 10 h after SAV administration, may indicate incomplete renal protection and a potential risk for the development of chronic kidney injury, requiring future follow-up.

## Highlights

• High levels of kidney injury biomarkers, such as uNGAL and KIM-1, have suggested incomplete renal recovery.
• Higher serum levels of angiopoietin-2 and vascular cell adhesion molecule-1 (VCAM-1) were observed in bleeding patients.
• VCAM-1 levels were correlated with kidney biomarkers, indicating potential endothelial damage alongside renal dysfunction.
• After antivenom: High levels of kidney biomarkers may point to incomplete renal protection and chronic kidney disease risk.

## Graphical abstract

**Figure:**
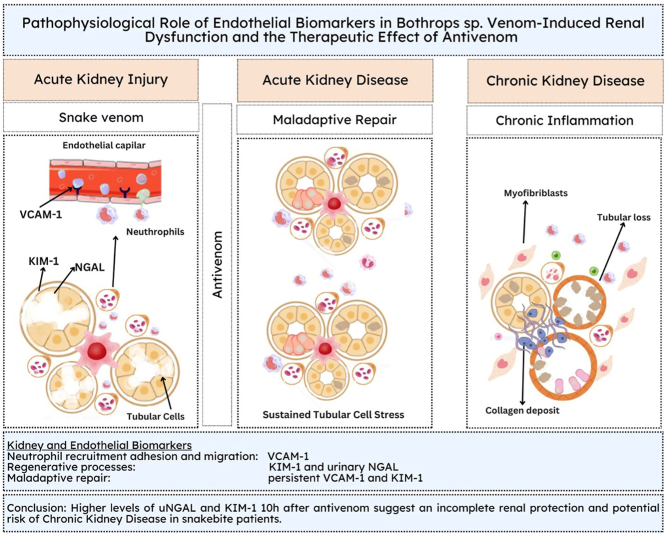

## 1 Introduction

Snakebite envenoming is a neglected public health problem in tropical and subtropical countries (Gutiérrez et al., 2016; Seifert et al., 2022). Underreporting of cases omits the real impact these envenomings have worldwide. Still, epidemiological data demonstrate that annually, there are 5.4 million incidents, 1.8 to 2.7 million envenomings, 81,000 to 138,000 deaths, and 400,000 cases of permanent sequelae (da Silva et al., 2023; Gutiérrez et al., 2016).

In Latin America, the genus *Bothrops* spp., which belongs to the family *Viperidae*, is the main snake responsible for snakebite envenoming. *Bothrops* spp. venom comprises various molecules such as metalloproteases (SVMPs), serine proteases (SVSPs), and phospholipases A_2_ (PLA_2_s), among others, which are responsible for hemotoxic, nephrotoxic, and proteolytic effects (Albuquerque et al., 2019; Cavalcante et al., 2023; da Silva et al., 2023; Mota et al., 2021). After envenoming, individuals can present with signs and symptoms, including pain, edema, blisters, myonecrosis, vascular lesions, coagulation disturbances, etc. (Cavalcante et al., 2023; Sgrignolli et al., 2011) Thrombotic microangiopathy and acute kidney injury (AKI) are significant complications secondary to these envenoming, occurring in 1.4–38.5 % of cases, commonly being oliguric, severe, early, and potentially lethal (Albuquerque et al., 2019, 2020; Mota et al., 2020).

Acute kidney injury is a complex and multifactorial syndrome characterized by a broad-spectrum pathophysiology influenced by the patient's characteristics and the clinical conditions of the injury (Rodrigues and Endre, 2023; Stanski et al., 2023). Many causes are associated with snake venom, such as coagulopathies, direct nephrotoxic action, and indirect effects possibly related to hemodynamic events and myoglobinuria (Albuquerque et al., 2020; Seifert et al., 2022). Endothelial injuries are believed to play a significant role in the pathogenesis of AKI. Venom-induced consumption coagulopathy, the most common and relevant systemic effect of snake envenoming, thrombotic microangiopathy, and intravascular coagulation can compromise blood flow, trigger ischemic mechanisms, increase inflammation, and induce oxidative stress via blood components. (Albuquerque et al., 2019; Cavalcante et al., 2023; Seifert et al., 2022; Wedasingha et al., 2020). Snakebite-associated thrombotic microangiopathy (TMA) is reported in a wide variety of different snake species, causing venom-induced consumption coagulopathy (VICC), which have different procoagulant toxins that target different clotting factors (Noutsos and Isbister, 2023). Despite these multiple candidate mechanisms for TMA in snakebite, further research is required to elucidate its underlying pathophysiology (Noutsos and Isbister, 2023).

Very few longitudinal studies have investigated the association between kidney dysfunction and coagulopathy in *Bothrops* envenoming, including potential changes following antivenom infusion (Albuquerque et al., 2019; Wen et al., 2023). Biomarkers of kidney injury, such as neutrophil gelatinase-associated lipocalin (NGAL) and kidney injury molecule 1 (KIM-1), have been promising in the evaluation of AKI, as well as endothelial biomarkers such as angiopoietin-1 (Ang-1), which has been suggested as a good predictor of AKI in *Bothrops* envenoming (Mota et al., 2021; Stanski et al., 2023).

Antivenoms remain the gold standard for the treatment of snake envenoming (Noutsos and Isbister, 2023). Whilst antivenom is the most valuable therapy for snakebite envenoming, evidence in support of the effectiveness of antivenom in treating venom-induced consumption coagulopathy is explicitly available for only a limited number of snake species (Noutsos and Isbister, 2023). The present study aims to investigate the effects of antivenom in renal and endothelial biomarkers abnormalities associated with spontaneous bleeding following *Bothrops* envenoming. Given the challenging management of complications related to snakebite envenoming, these new biomarkers might explain the relation between coagulopathies, kidney function, predicting the development of chronic kidney dysfunction (da Silva et al., 2023; Yoon et al., 2022).

## 2 Materials and methods

### 2.1 Study design and patients

This is an observational, longitudinal, and prospective study with 34 victims of *Bothrops* snakebite admitted to a reference hospital for emergency care in Ceará State (Brazil) between August 2019 and November 2020. The study included patients over 18 years old of both genders who signed the free and informed consent form and suffered *Bothrops* snakebites confirmed by direct presentation of the snake to the clinical staff or photographic evidence of the animal, and consistent clinical, epidemiological and laboratory data based on the guidelines of the Brazilian Ministry of Health (Fundação Nacional de Saúde, 2001) and of the this hospital (Governo do Estado do Ceará, 2021).

All patients were treated with appropriate antivenom within 8 h of admission. The specific treatment of snakebite patients was based on the guidelines of the Brazilian Ministry of Health, and it is distributed to public and private health units without local costs. The presence of hemorrhagic symptoms and coagulation abnormalities in laboratory tests is one of the criteria used to define the envenoming due to *Bothrops* snakes, and the severity of patients according to the Brazilian Ministry Guideline (Fundação Nacional de Saúde, 2001). These signals are compulsorily examined and asked for by healthcare workers for every patient. In the current study, the presence of bleeding was considered. Brazilian experience in the organization of a program for snakebite envenoming surveillance ensures the availability to all patients in the country (Fan and Monteiro, 2018).

Patients hospitalized for longer than 8 h after the incident, those who received antivenom before the first sample collection, pregnant patients, and those diagnosed with diabetes mellitus, systemic arterial hypertension, acute or chronic kidney injury, or who were using diuretics, and cases where it was not possible to confirm the *Bothrops* snakebite envenoming were excluded. The snake species was most *Bothrops erythromelas*, which is well adapted in Ceará, Northeast Brazil, with a wide distribution (Ministério da Saúde, 2024). This species was identified through the animal's photo or examination, and the patient's clinical picture associated with the envenoming.

*Bothrops* is the genus that is associated with incoagulation and bleeding events, and these are associated with complications, organ damage, and death (Cavalcante et al., 2023). Once *Bothrops erythromelas* is highly associated with coagulation abnormalities and hemorrhagic symptoms, and this species is commonly found in the state of Ceará (Ministério da Saúde, 2024), patients were divided according to the presence of spontaneous bleeding upon hospital admission. Spontaneous bleeding was defined as any signs of hemorrhage visible on physical examination, such as gingival, urinary, cutaneous, or ocular bleeding, or detected by the radiologist associated with a drop in hemoglobin on hospital admission.

### 2.2 Sample collection and assessed parameters

Sociodemographic and clinical data were collected during the patient's hospitalization using DATATOX (Brazilian Poisoning Data System) and SINAN (Information System of Notifiable Diseases). Blood and urine samples were collected three times: before antivenom infusion (C1), 10 h after antivenom infusion (C2), and 20 h after antivenom infusion (C3).

Hematological parameters (hemoglobin, hematocrit, leukocytes, erythrocytes, and platelets) were analyzed using the ADVIA® 2120i hematological analyzer (Siemens Healthineers). Coagulation parameters (Prothrombin time - PT, activated partial thromboplastin time - aPTT) were processed using the Automatic Sysmex® CA-1500 analyzer.

Biochemical urine parameters (serum urea, serum creatinine) were analyzed with CMD 800i (Wiener Lab), and plasma levels of sodium (Na^+^) and potassium (K+) with the Electrolyte Analyzer 9180 (Roche®), expressed in mEq/L. Additional urine samples were spun for 5 min at 1500 rpm to analyze hematuria, proteinuria, and urine density. Samples were then aliquoted into identified Eppendorf's for storage at −80 °C until biomarker measurement.

Upon admission, patients were physically examined to determine whether they had hemorrhagic syndrome, characterized by active bleeding, such as at the snakebite site, gingival bleeding, and others. From this, it was possible to divide patients into a “bleeding group” and a “non-bleeding group.” In addition, patients who presented PT > 120 s and aPPT >180 s were classified as “incoagulable”.

### 2.3 Definition of acute kidney injury

Kidney function was analyzed based on daily serum creatinine (sCr) measurements during hospitalization, considering the lowest value from admission to discharge as the baseline sCr Glomerular filtration rate (GFR) was calculated using the 2021 Chronic Kidney Disease Epidemiology Collaboration (CKD-EPI) equation (Kidney Disease: Improving Global Outcomes CKD Work Group, 2024). AKI classification followed the sCr of Kidney Disease Improving Global Outcomes (sCr-KDIGO) criteria: a creatinine increase of at least 1.5 times the baseline or an increase of ≥0.3 mg/dl (≥26.5 μmol/l) as compared to baseline (Khwaja, 2012).

### 2.4 Quantification of systemic biomarkers

Biomarkers were measured in serum and urine samples obtained at times C1, C2, and C3. The assays for measurements were based on enzyme-linked immunosorbent assay (ELISA) using specific kits for each biomarker. The procedures were followed according to the manufacturer instructions for Angiopoietin-1 (Ang-1, DY923, R&D Systems®), Angiopoietin-2 (Ang-2, DY623, R&D Systems®), Syndecan-1 (ab47352, Abcam®), Vascular Cell Adhesion Molecule 1 (VCAM-1, ab47355, Abcam®) and Neutrophil Gelatinase-Associated Lipocalin (NGAL, DY1757, R&D Systems®) in serum, and for NGAL, Kidney Injury Molecule-1 (KIM-1, DY1750B, R&D Systems®) and Monocyte Chemoattractant Protein-1 (MCP-1, DY279, R&D Systems®) in urine.

### 2.5 Statistical analysis

Patients were analyzed according to their hemorrhagic status (“bleeding” vs “non-bleeding”). Categorical variables were presented as absolute count and relative frequency in percentages. Associations between categorical variables were assessed using chi-square or Fisher's exact tests. Continuous variables were assessed for normality using the Shapiro-Wilk test, Q-Q graphs, histograms, and dispersion measures. Normal data were presented as mean ± standard deviation, non-normal data as median, and interquartile range. Student's t-test or Mann-Whitney test compared two independent groups. Correlation analyses between continuous data used Spearman or Pearson's correlation (p < 0.05 Data were analyzed with SPSS (version 23, IBM Corp.) and JMP Pro version 17 (SAS Institute, USA). Repeated measures mixed model analysis (JMP Pro version 17, SAS Institute, USA) was performed to analyze longitudinal measurements of each kidney and endothelial biomarker according to patient bleeding status and time points as fixed effects, with each patient included as a random effect. Least square means plots with confidence limits were generated from that analysis. Similar mixed models were performed using kidney biomarker trajectories as the outcome and including the first measurement of each endothelial marker as a fixed effect to assess if the first measurement of each endothelial marker could associate with the trajectory the kidney biomarker would later develop.

## 3 Results

**Fig. 1:**
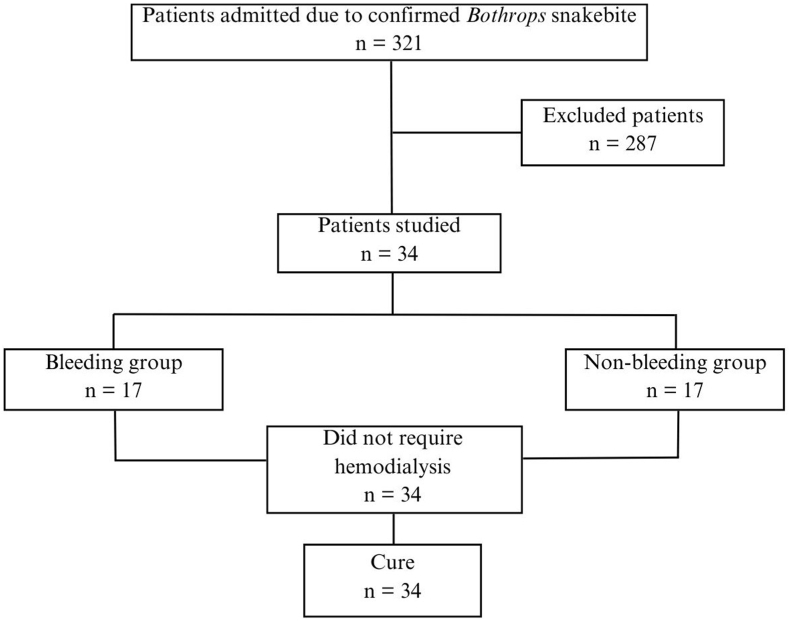
Study flowchart.

Three hundred twenty-one patients were admitted to the tertiary hospital due to *Bothrops* snakebite. Of these individuals, 287 were excluded because they had received antivenom before the first urine and blood collection time, were under 18 years old, were admitted to the health unit more than 8 h after the envenoming or had any other exclusion criteria. All patients recovered properly, and none required hemodialysis (Fig. 1).

Thus, 34 patients were assessed. Among these, 32 species were identified as *Bothrops erythromelas*, 1 as *Bothrops bilineatus* and 1 as *Bothrops jararaca*. The majority were men, with a mean of 46.3 years and 50 % presenting spontaneous bleeding upon admission (Table 1). It is important to highlight that the skin or mucosal bleeding was evaluated upon hospital admission; however, the spontaneous bleeding may have occurred before and this sign was not considered in patient classification.

**Table 1:** Sociodemographic and laboratory parameters of patients admitted after *Bothrops* envenoming according to the presence of hemorrhagic syndrome.

	Total group (n = 34)	Bleeding		
		No (n = 17)	Yes (n = 17)	p
**Age (Years)**	46.3 ± 14.5	44.1 ± 16.6	47.5 ± 12.3	0.502
**Sex**				0.656
Male	28.0 (82)	15.0 (88.2)	13.0 (76.5)	
Female	6.0 (18)	2.0 (11.8)	4.0 (23.5)	
**Local**				0.290
Rural	21.0 (61.8)	9.0 (52.9)	12.0 (70.6)	
Urban	13.0 (38.2)	8.0 (47.1)	5.0 (29.4)	
**Post bite (hours)**	5.8 ± 1.7	5.6 ± 2.3	5.9 ± 0.9	0.675
**Renal assessment**				
**AKI (sCr KDIGO criteria)**				0.730
Yes	19.0 (56)	9.0 (52.9)	10.0 (58.8)	
No	15.0 (44)	8.0 (47.1)	7.0 (41.2)	
**Serum creatinine (mg/dL)**	1.1 ± 0.3	1.2 ± 0.3	1.1 ± 0.3	0.535
**Estimated GFR (mL/min/1.73m^2^)**	88.1 ± 27.8	90.4 ± 27.8	87.3 ± 28.8	0.752
**Serum urea (mg/dL)**	38.2 ± 10.3	38.1 ± 10.8	37 ± 8.4	0.752
**Urine Protein/creatinine ratio (PCR)**	0.75 (0.37–1.32)	0.47 (0.27–0.75)	1.09 (0.79–2.05)	**0.034**
**Albuminuria/creatinine ration (ACR) (mg/g Cr)**	0.50 (0.30–0.70)	0.5 (0.30–0.70)	0.5 (0.30–0.70)	0.736
**Other parameters**				
**Serum glucose (mg/dL)**	106.8 ± 19.4	99.2 ± 15.5	109.8 ± 21.3	0.297
**Serum sodium (mmol/L)**	140.4 ± 2.5	139.6 ± 2.2	141.1 ± 2.7	0.085
**Serum chloride (mEq/L)**	103.3 ± 2.9	102.0 ± 2.4	104.3 ± 3.1	0.082
**Total serum calcium (mg/dL)**	9.5 ± 0.5	9.7 ± 0.6	9.4 ± 0.5	0.334
**Hemoglobin (g/dL)**	14.1 ± 0.9	14.3 ± 0.9	14 ± 1	0.284
**Hematocrit (%)**	42.4 ± 3	43 ± 2.5	42 ± 3.4	0.328
**Blood leukocytes (mm^3^)**	11561.7 ± 2946.4	11615.9 ± 2588.8	11624.7 ± 3389.7	0.993
**Blood platelets (mm^3^)**	181314 ± 95313.0	211117 ± 83499.0	151764 ± 102274.0	0.073
**Coagulation**				**0.034**
Coagulable	7 (21)	6 (35.3)	1 (5.9)	
Incoagulable∗	27 (79)	11 (64.7)	16 (94.1)	
**TP (s)**	121 (20.7–121)	121 (22.1–121)	121 (0–121)	0.736
**aPTT (s)**	181 (34.5–181)	181 (37.4–181)	180 (29.9–181)	0.322

On admission, sociodemographic factors showed no significant differences between bleeding and non-bleeding groups in most laboratory parameters, including electrolytes, hematological parameters, and coagulation tests (Table 1). However, the bleeding group had significantly higher proteinuria levels on admission. No significant differences were observed in serum creatinine and urea levels, estimated glomerular filtration rate (GFR), or albuminuria. According to serum creatinine of Kidney Disease Improving Global Outcomes (sCr-KDIGO) criteria (sCr-KDIGO criteria), 19 patients had AKI, and of these, 17 were in stage 1 (Table 1).

Categorical data are expressed as absolute counts and percentages in parentheses. Quantitative data is expressed as mean ± standard deviation or median and interquartile range in parentheses. GFR: glomerular filtration rate. PT: Prothrombin time. aPPT: Activated partial thromboplastin time. Reference Values: Hemoglobin 11.5–18 g/dL; Hematocrit 36–54 %; Platelets 150,000–450,000 mm^3^; leukocytes 3600–10,000 mm^3^; Creatinine 0.6–1.3 mg/dL; Urea 13–43 mg/dL; Sodium 135–146 mmol/L; Potassium 3.5–5.3 mEq/L; Glucose 70–100 mg/dL; Calcium 8.5–10.5 mg/dL ∗Patients who presented PT > 120 s and aPPT >180 s.

**Fig. 2:**
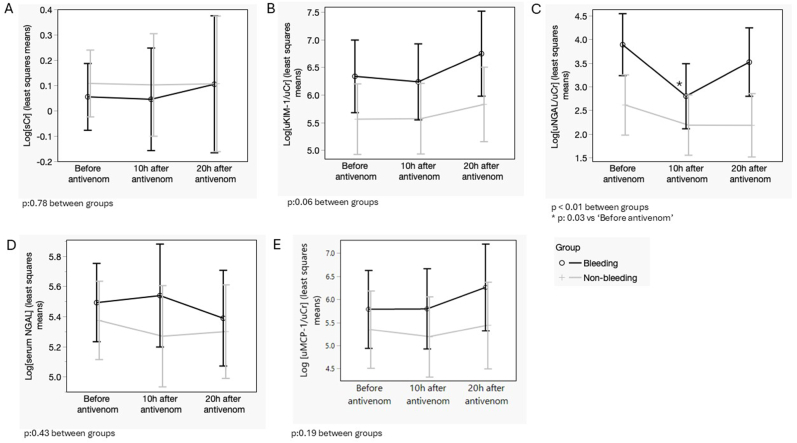
Trajectories of kidney functional and damage biomarkers over the three-time points assessed by spontaneous bleeding status at hospital admission. (a) Serum creatinine; (b) uKIM-1/uCr; (c) uNGAL/uCr; (d) serum NGAL; (e) uMCP-1/uCr. Graphs depict least squares means and confidence limits, and variables were log-transformed when residuals were not normally distributed in each model.

By the similar incidence of sCr-based AKI in both bleeding and non-bleeding groups, the trajectories of sCr over the three collection time points were not different between groups (Fig. 2A). However, the kidney damage biomarkers uKIM-1/uCr, uNGAL/uCr, and uMCP-1/uCr increased, mainly in the bleeding group (Fig. 2B, C, and 2E), regardless of antivenom infusion. Improvement of kidney function was suggested up to 10h of antivenom infusion in the bleeding group when uNGAL/uCr was assessed (Fig. 2C). From 10h after antivenom infusion, the serum level of NGAL decreased (Fig. 2D). Urine MCP-1/uCr increased in all groups, but there were no differences (Fig. 2D and E).

**Fig. 3:**
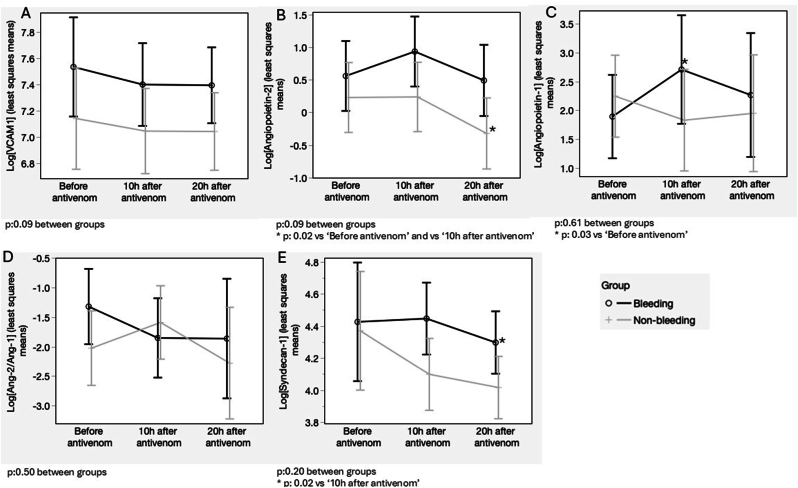
Trajectories of endothelial biomarkers were assessed over the three time points by spontaneous bleeding status at hospital admission. (a) VCAM-1; (b) Ang-2; (c) Ang-1; (d) Ang-2/Ang-1 ratio; (e) Syndecan-1. Graphs depict least squares means and confidence limits, and variables were log-transformed when residuals were not normally distributed in each model.

Over the time of antivenom infusion, the serum level of VCAM-1, Ang-2, and Syndecan-1 decreased in both groups, but there were no differences between them in this cohort (Fig. 3A, B, and 3E). Ang-1 increased in the bleeding group after antivenom infusion, significantly differing from no-bleeding, decreasing after 10h (Fig. 3C). Ang-2/Ang-1 ratio (Fig. 3D) did not present significant differences in this cohort.

**Fig. 4:**
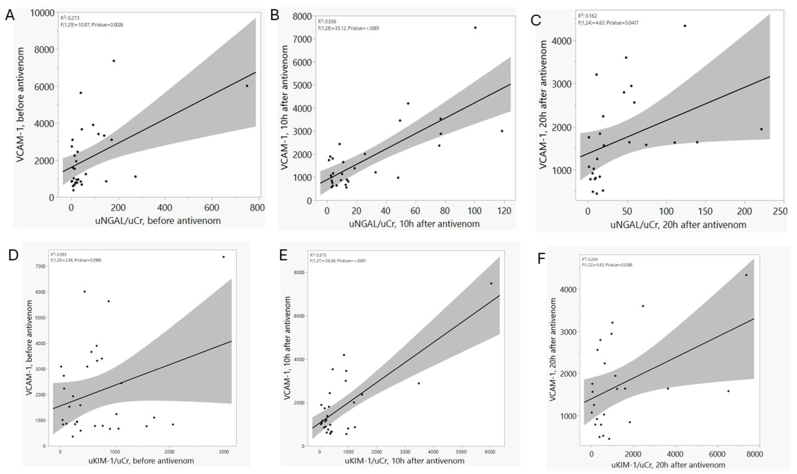
Correlation between VCAM-1 and kidney damage biomarkers at each time point. (a) VCAM-1 vs. uNGAL/uCr before antivenom treatment; (b) VCAM-1 vs. uNGAL/uCr 10h after antivenom treatment; (c) VCAM-1 vs uNGAL/uCr 20h after antivenom treatment; (d) VCAM-1 vs uKIM-1/uCr before antivenom treatment; (e) VCAM-1 vs uKIM-1/uCr 10h after antivenom treatment; F. VCAM-1 vs uKIM-1/uCr 20h after antivenom treatment.

Serum levels of VCAM-1 and Ang-2 positively correlated with uNGAL/uCr and uKIM-1/uCr levels at each timepoint, especially after treatment with antivenom (Fig. 4 and Fig. S1), and serum levels of Ang-1 positively correlated with uNGAL/uCr, but not with uKIM-1/uCr levels at each timepoint (Fig. S2). Serum levels of Ang-1 and Ang-2 positively correlated with VCAM-1 levels at each timepoint, especially in the bleeding group for Ang-2 (Fig. S3).

The best correlations between endothelial and kidney biomarkers in this cohort were VCAM-1 and uNGAL/uCr (Fig. 4B) and VCAM-1 and uKIM-1/uCr (Fig. 4E), both 10h after antivenom infusion. Other comparisons are in the supplementary material.

However, initial levels of serum Ang-1, Ang-2, and VCAM-1 were associated with the later trajectories of uNGAL/uCr at the mixed model analysis (Table 2) but not with uKIM-1/uCr (Table S1).

**Table 2:** Comparisons of uNGAL/uCr levels before antivenom, 10h, and 20h after antivenom extracted from linear mixed models in which fixed effects included bleeding, timepoints, the bleeding by time interactions, and different endothelial markers measured before antivenom.

Outcome: uNGAL/uCr trajectory	Fixed effects	F ratio	p-value
Model 1	Bleeding	4.06	0.054
	**Timepoints**	**6.43**	**0.004**
	Bleeding by time interaction	2.01	0.150
	**VCAM-1 level before antivenom**	**8.76**	**0.006**
Model 2	**Bleeding**	**10.61**	**0.003**
	**Timepoints**	**5.58**	**0.008**
	Bleeding by time interaction	2.10	0.139
	**Ang-1 level before antivenom**	**7.81**	**0.009**
Model 3	**Bleeding**	**9.10**	**0.005**
	**Timepoints**	**6.11**	**0.005**
	Bleeding by time interaction	2.30	0.115
	**Ang-2 level before antivenom**	**9.98**	**0.004**

Ang-1: Angiopoietin-1. Ang-2: Angiopoietin-2. uCr: Urinary creatinine. uNGAL: Urinary neutrophil gelatinase-associated lipocalin. VCAM-1: vascular cell adhesion molecule-1.

## 4 Discussion

This is the first longitudinal study evaluating the association between hemorrhagic manifestations in patients who suffered *Bothrops* snakebite and the course of kidney injury and endothelial biomarkers before and after antivenom infusion. The epidemiological profile of our population agrees with the literature that male individuals of economically active age, involved with agricultural activities, are commonly involved in *Bothrops* snakebite. (da Silva et al., 2023).

*Bothrops* venom components, particularly metalloproteinases, and phospholipases, induce local tissue damage and systemic coagulopathy. (Thomazini et al., 2021). Studies have highlighted the role of coagulation abnormalities in renal damage and microvascular endothelial lesions that favor AKI manifestation. (Albuquerque et al., 2020; Mota et al., 2021). This study revealed that 50 % of patients presented with spontaneous bleeding upon admission. Hemotoxic and proteolytic characteristics of *Bothrops* venom commonly cause hemorrhagic events (Albuquerque et al., 2020). Coagulation abnormalities, the venom's direct toxicity, immunologic mechanisms, and inflammatory response were pointed as essential causes of tubular and glomerular damage, which may explain elevated proteinuria before antivenom infusion, mainly in the presence of hemorrhagic syndrome (Albuquerque et al., 2020; Mota et al., 2021; Pinho et al., 2008).

The correct classification of the AKI subtype, using new kidney biomarkers, is fundamental and allows a better approach for the patient once they can detect abnormalities earlier than serum creatinine elevation (Rodrigues and Endre, 2023). This study suggests that patients with spontaneous bleeding at the hospital admission might be experiencing, at least, subclinical AKI (also recently called AKI stage 1S, where tubular injury biomarkers are positive and serum creatinine, as well as urine output, is expected) as demonstrated by increase uKIM-1/uCr and uNGAL/uCr levels both before and after the treatment with anti-venom (Ostermann et al., 2020). uNGAL and uKIM-1 are well-established markers as early and sensitive indicators of tubular injury in the course of AKI (Szumilas et al., 2024).

Urinary biomarkers slopes during kidney damage represent a dynamic mechanistic pathway, which may provide insights to improve patient management (Wen et al., 2023). The increase of some urinary biomarkers 10h after antivenom infusions, such as urinary Neutrophil Gelatinase-Associated Lipocalin (uNGAL), urinary Monocyte Chemotactic Protein-1 (MCP-1), and Kidney Injury Molecule 1 (KIM-1), deserves attention. Urinary NGAL is a 25-kDa protein produced by tubular cells in the kidney, and its receptors are multifactorial (Romejko et al., 2023). It is a very sensitive marker of ischemic injury and can be detected in plasma within 2 h of AKI, with a concentration peak after 6 h (Mishra et al., 2003). Increased serum NGAL levels were observed for approximately five days after AKI before they decreased (Romejko et al., 2023). This fact contributes to the importance of following urinary NGAL levels for the long term. KIM-1, a multifunctional glycoprotein and biological marker, belongs to the T-cell immunoglobulin and mucin domain family (TIM) of proteins, which are presented on the immune cells and participate in the regulation of immune reactions (Karmakova et al., 2021). Some KIM-1 domains serve as a signal for macrophages and epithelial cells to absorb dying cells, performing the function of scavenger receptors mediating *in situ* elimination of cellular debris in case of tissue damage. (DeKruyff et al., 2010; Günther and Seyfert, 2018). In ischemic or toxic kidney injury, activation of KIM-1 synthesis in the cells of the damaged tubules and its increased expression on the apical cell membrane is observed (Karmakova et al., 2021). On the other hand, the increase of KIM-1 production in the proximal tubular cells in AKI is likely to be an adaptive response, being not only the marker of injury but the reflection of the reparation activity (Zhang et al., 2008). Urinary MCP-1 is secreted by mononuclear leukocytes, cortical tubular epithelial cells, and podocytes in the kidney and is associated with renal inflammation, glomerular damage, and tubular atrophy, mainly in proximal and distal convoluted tubules (Albuquerque et al., 2019). The increase of these biomarkers after 10h, more intense in bleeding patients, may reveal tubular maladaptive repair, consistent with animal studies (Zheng et al., 2021), exacerbated by renal inflammation via biomarkers, such as MCP-1 production, which could contribute to AKI-CKD transition.

Many studies have reported the capability of early delivery of antivenom in preventing negative outcomes in snakebite envenoming (Khimmaktong et al., 2022). The slight increase of KIM-1 and MCP-1 after antivenom infusion needs to be followed in snakebite patients to explain if it is possible that the antivenom could trigger a renal inflammatory response by the presence of heterologous proteins, which are not intense, once other biomarkers, such as serum NGAL, decrease significantly after antivenom infusion. Animal studies could be designed to determine if these kidney abnormalities are related to venom or antivenom. From a perspective of endothelial biomarkers, the decrease of Syndecan-1 after antivenom infusion in all groups of patients suggests the repair of the glycocalyx layer. (Hahn et al., 2021). Sustained tissue injury and inflammation, and slower restoration of tubular health are associated with a higher risk of kidney disease progression (Wen et al., 2023). The antivenom administered does not contain the *Bothrops erythromelas* or *leucurus* toxins, common species reported in Ceara, Northeast Brazil, which could reduce its action in this envenoming (Butantan, 2022). Thus, we suggest kidney evaluation of these patients with uKIM-1/uCr and mostly uNGAL/uCr for early detection of renal injuries by aiding in renal protection and tubular epithelial cell repair (Albuquerque et al., 2019; Ostermann et al., 2020; Senthilkumaran et al., 2019). The association between thrombotic microangiopathy (TMA) and acute kidney injury related to *Bothrops* venom has been reported (Mota et al., 2020). However, there is no clear evidence for antivenom preventing the development of TMA in patients presenting with venom-induced consumption coagulopathy (VICC), and existing data are both limited and/or confounded, including by treatment bias and interrelated variables such as time to hospitalization and access to best supportive care (Noutsos et al., 2022). Moreover, there are no interventional randomized controlled trials in snakebite-associated envenoming, and antivenom remains the standard of care for patients with snake envenoming (Noutsos and Isbister, 2023). Otherwise, a wide range of clinical toxin syndromes related to VICC, with partial and complete presentations, contribute to many biases.

The correlation between systemic envenoming and the levels of serum biomarkers depicts how venom acts and could be a potential predictor of complications (Wedasingha et al., 2024). A possible increase in Ang-2 levels in the bleeding group might indicate a greater need for endothelial repair due to more severe vascular damage (Saharinen et al., 2008). Ang-1 is a protective protein for blood vessels, promoting tissue remodeling and angiogenesis as a compensatory mechanism for vascular repair (Mota et al., 2021). On the other hand, Ang-2 is an inhibitor of Ang-1 repair actions, and its imbalance was evident in the bleeding group. Levels of Ang-1 and Ang-2 were correlated with VCAM-1 levels, especially in patients with spontaneous bleeding at admission for Ang-2. Ang-2 is upregulated after endothelial activation in inflammatory situations and can activate VCAM-1 expression in endothelial cells, which increases leukocytes' adhesion to the endothelial surface and promotes further damage (Li et al., 2020). Some studies compared serum NGAL and clusterin concentration (sClu) with secretory phospholipase A_2_ (sPLA_2_), showing sPLA_2_ activity as an early predictor of systemic envenoming following snakebites, particularly in Russel's viper bites (Wedasingha et al., 2024).

Kidney injury due to snakebite envenoming is intrinsically related to endothelial damage, as scientific literature has reported (Mota et al., 2021). Increased levels of Ang-1, Ang-2, and VCAM-1 were correlated with the degree of kidney injury determined by kidney damage biomarkers. Accordingly, patients with spontaneous bleeding had slightly higher levels of VCAM-1 and Ang-2 during the study period than non-bleeding patients. The intense interaction between urinary NGAL and the level of VCAM-1, Ang-1, and Ang-2 before antivenom administration in linear mixed models highlights the importance of repairing the endothelial to overcome tubular and functional renal dysfunction (He et al., 2021).

As in other settings of acute kidney injuries, patients who suffered snakebite envenoming are at risk of subsequent chronic kidney disease (CKD) after initial recovery, and long-term follow-up is strongly recommended (Noutsos et al., 2022). Given the current classification of acute kidney injury as subclinical AKI based on non-traditional biomarkers and the increase in endothelial biomarkers in patients with hemorrhagic syndrome, we suggest investing in studies to provide endothelial protection therapy as a complementary treatment to antivenom to reduce the incidence of AKI secondary to *Bothrops* envenomation. In the context of pulmonary hypertension, for example, erythropoietin and sildenafil may be promising drugs for protecting endothelial cells, decreasing the dysfunction of these cells after hypoxia, which is an event that can occur in AKI after *Bothrops* snakebite in ischemia/reperfusion (I/R) injury (Albuquerque et al., 2020; Gammella et al., 2013).

Our study has limitations. The small sample size prevented us from conducting a multivariate analysis, limiting the robustness and generalizability of our findings. We could not quantify venom components in the biological samples, which could directly influence kidney and endothelial biomarkers.

## 5 Conclusions

In conclusion, endothelial damage resulting from *Bothrops* envenoming contributes to kidney injury that persists despite antivenom administration. Likely, the snake venom toxins fixed into kidney cells may escape neutralization by antivenom antibodies (Butantan, 2022). Our findings underscore the complexity of the pathophysiological mechanisms involved, highlighting that antivenom alone may not fully mitigate renal damage. This study emphasizes the need for additional studies addressing therapeutic strategies to repair vascular integrity and reduce kidney injury secondary to *Bothrops* snakebites. In addition, these patients' follow-up to evaluate renal function after the hospitalization period should be recommended.

## CRediT authorship contribution statement

**Nicole Coelho Lopes:** Writing – review & editing, Writing – original draft, Visualization, Validation, Supervision, Project administration, Methodology, Data curation, Conceptualization. **Gdayllon Cavalcante Meneses:** Writing – original draft, Supervision, Project administration, Methodology, Formal analysis, Data curation, Conceptualization. **Ranieri Sales de Souza Santos:** Validation, Supervision, Methodology, Investigation. **Leticia Machado de Araújo:** Validation, Investigation. **Bruna Viana Barroso Martins:** Validation, Investigation. **Katarina Maria dos Reis Araújo:** Investigation. **Valéria Holanda Nogueira de Aquino:** Investigation. **Igor Moreira de Almeida:** Investigation. **Sandra Mara Brasileiro Mota:** Visualization, Supervision, Methodology, Investigation, Conceptualization. **Geraldo Bezerra da Silva Junior:** Writing – review & editing, Resources, Methodology, Funding acquisition. **Camila Eleuterio Rodrigues:** Writing – review & editing, Formal analysis. **Elizabeth De Francesco Daher:** Resources, Funding acquisition. **Polianna Lemos Moura Moreira Albuquerque:** Writing – review & editing, Data curation, Conceptualization. **Alice Maria Costa Martins:** Writing – review & editing, Visualization, Validation, Resources, Methodology, Funding acquisition, Conceptualization.

## Informed consent statement

Informed consent was obtained from all subjects involved in the study. Patient identities were protected through data anonymization.

**Table:** Abbreviations

ACR	Albuminuria/creatinine ratio
AKI	Acute kidney injury
Ang-1	Angiopoietin-1
Ang-2	Angiopoietin-2
aPTT	Activated partial thromboplastin time
CKD	Chronic Kidney Disease
CKD-EPI	Chronic Kidney Disease Epidemiology Collaboration
ELISA	Enzyme-linked immunosorbent assay
GFR	Glomerular filtration rate
I/R	Ischemia/reperfusion
K^+^	Ion potassium
KDIGO	Kidney Disease: Improving Global Outcomes
KIM-1	Kidney injury molecule 1
MCP-1	Monocyte Chemoattractant Protein-1
Na^+^	Ion sodium
NGAL	Neutrophil gelatinase-associated lipocalin
PCR	Urine protein/creatinine ratio
PLA_2_s	Phospholipase A2
PT	Prothrombin time
SAV	Snakebite antivenom
sClu	Serum clusterin
sCr	Serum creatinine
sNGAL	Serum neutrophil gelatinase-associated lipocalin.
SVMPs	Metalloproteases
SVSPs	Serine proteases
TIM	T-cell immunoglobulin and mucin domain family
TMA	Thrombotic microangiopathy
uCr	Urinary creatinine
uKIM-1	Urinary kidney injury molecule 1
uMCP-1	Urinary monocyte Chemoattractant Protein-1
uNGAL	Urinary neutrophil gelatinase-associated lipocalin.
VCAM-1	Vascular Cell Adhesion Molecule 1
VICC	Venom-induced consumption coagulopathy

## Ethics statement

The study was conducted according to the guidelines of the Declaration of Helsinki and approved by the University of Fortaleza Ethics Committee (CAAE: 41664214.5.0000.5052) in 2019.

## Funding

This research was supported by Fundação Cearense de Apoio ao Desenvolvimento Científico e Tecnológico (Funcap) [grant number: BMD-0008-02044.01.05/22]; Conselho Nacional de Desenvolvimento Científico e Tecnológico (10.13039/501100003593CNPq) [grant numbers: 405963/2016-5; 301174/2017-2; and 130280/2021-6]; and10.13039/501100001803Charles Darwin University.

## Declaration of competing interest

The authors declare the following financial interests/personal relationships which may be considered as potential competing interests:Nicole Coelho Lopes reports financial support was provided by Foundation for Scientific and Technological Development and Support of Ceará. Ranieri Sales de Souza Santos reports financial support was provided by 10.13039/501100003593National Council for Scientific and Technological Development. Geraldo Bezerra da Silva reports a relationship with 10.13039/501100003593National Council for Scientific and Technological Development that includes: funding grants. Elizabeth De Francesco Daher reports a relationship with 10.13039/501100003593National Council for Scientific and Technological Development that includes: funding grants. If there are other authors, they declare that they have no known competing financial interests or personal relationships that could have appeared to influence the work reported in this paper.

## Data Availability

Data will be made available on request.

## References

[bib1] Albuquerque P., da Silva Junior G., Meneses G., Martins A., Lima D., Raubenheimer J., Fathima S., Buckley N., Daher E. (2019). Acute kidney injury induced by Bothrops venom: insights into the pathogenic mechanisms. Toxins.

[bib2] Albuquerque P.L.M.M., Paiva J.H.H.G.L., Martins A.M.C., Meneses G.C., Silva Júnior G.B. da, Buckley N., Daher E.D.F. (2020). Clinical assessment and pathophysiology of Bothrops venom-related acute kidney injury: a scoping review. J. Venom. Anim. Toxins Incl. Trop. Dis..

[bib3] Butantan I. (2022).

[bib4] Cavalcante J.S., de Almeida D.E.G., Santos-Filho N.A., Sartim M.A., de Almeida Baldo A., Brasileiro L., Albuquerque P.L., Oliveira S.S., Sachett J.A.G., Monteiro W.M., Ferreira R.S. (2023). Crosstalk of inflammation and coagulation in Bothrops snakebite envenoming: Endogenous signaling pathways and pathophysiology. Int. J. Mol. Sci..

[bib5] da Silva W.R.G.B., de Siqueira Santos L., Lira D., de Oliveira Luna K.P., Fook S.M.L., Alves R.R.N. (2023). Who are the most affected by Bothrops snakebite envenoming in Brazil? A Clinical-epidemiological profile study among the regions of the country. PLoS Neglected Trop. Dis..

[bib6] DeKruyff R.H., Bu X., Ballesteros A., Santiago C., Chim Y.-L.E., Lee H.-H., Karisola P., Pichavant M., Kaplan G.G., Umetsu D.T., Freeman G.J., Casasnovas J.M. (2010). T cell/transmembrane, Ig, and mucin-3 allelic variants differentially recognize phosphatidylserine and mediate phagocytosis of apoptotic cells. J. Immunol..

[bib7] Fan H.W., Monteiro W.M. (2018). History and perspectives on how to ensure antivenom accessibility in the most remote areas in Brazil. Toxicon.

[bib8] Fundação Nacional de Saúde (2001). Manual de diagnóstico e tratamento de acidentes por animais peçonhentos.

[bib9] Gammella E., Leuenberger C., Gassmann M., Ostergaard L. (2013). Evidence of synergistic/additive effects of sildenafil and erythropoietin in enhancing survival and migration of hypoxic endothelial cells. Am. J. Physiol. Lung Cell. Mol. Physiol..

[bib10] Governo do Estado do Ceará (2021).

[bib11] Günther J., Seyfert H.-M. (2018). The first line of defence: insights into mechanisms and relevance of phagocytosis in epithelial cells. Semin. Immunopathol..

[bib12] Gutiérrez J.M., Escalante T., Rucavado A., Herrera C. (2016). Hemorrhage caused by snake venom metalloproteinases: a journey of discovery and understanding. Toxins.

[bib13] Hahn R.G., Zdolsek M., Zdolsek J. (2021). Plasma concentrations of syndecan‐1 are dependent on kidney function. Acta Anaesthesiol. Scand..

[bib14] He Y., Li H., Yao J., Zhong H., Kuang Y., Li X., Bian W. (2021). HO-1 knockdown upregulates the expression of VCAM-1 to induce neutrophil recruitment during renal ischemia-reperfusion injury. Int. J. Mol. Med..

[bib15] Karmakova T.A., Sergeeva N.S., Kanukoev K.Yu, Alekseev B.Ya, Kaprin A.D. (2021). Kidney injury molecule 1 (KIM-1): a multifunctional glycoprotein and biological marker. Sovrem Tehnol Med.

[bib16] Khimmaktong W., Nuanyaem N., Lorthong N., Hodgson W.C., Chaisakul J. (2022). Histopathological changes in the liver, heart and kidneys following Malayan pit viper (calloselasma rhodostoma) envenoming and the neutralising effects of hemato polyvalent snake antivenom. Toxins.

[bib17] Khwaja A. (2012). KDIGO clinical practice guidelines for acute kidney injury. Nephron Clin. Pract..

[bib18] Kidney Disease: Improving Global Outcomes (KDIGO) CKD Work Group (2024). KDIGO 2024 clinical practive guideline for the evauation and management of chronic kidney disease. Kidney Int..

[bib19] Li Z., Korhonen E.A., Merlini A., Strauss J., Wihuri E., Nurmi H., Antila S., Paech J., Deutsch U., Engelhardt B., Chintharlapalli S., Koh G.Y., Flügel A., Alitalo K. (2020). Angiopoietin-2 blockade ameliorates autoimmune neuroinflammation by inhibiting leukocyte recruitment into the CNS. J. Clin. Investig..

[bib20] Ministério da Saúde (2024).

[bib21] Mishra J., Ma Q., Prada A., Mitsnefes M., Zahedi K., Yang J., Barasch J., Devarajan P. (2003). Identification of neutrophil gelatinase-associated lipocalin as a novel early urinary biomarker for ischemic renal injury. J. Am. Soc. Nephrol..

[bib22] Mota S.M.B., Albuquerque P.L.M.M., Meneses G.C., da Silva Junior G.B., Martins A.M.C., De Francesco Daher E. (2021). Role of endothelial biomarkers in predicting acute kidney injury in Bothrops envenoming. Toxicol. Lett..

[bib23] Mota S.M.B., Albuquerque P.L.M.M., Silva Júnior G.B.D., Daher E.D.F. (2020). Thrombotic microangiopathy due to Bothrops erythromelas: a case report in Northeast Brazil. Rev. Inst. Med. trop. S. Paulo.

[bib24] Noutsos T., Currie B.J., Wijewickrama E.S., Isbister G.K. (2022). Snakebite associated thrombotic microangiopathy and recommendations for clinical practice. Toxins.

[bib25] Noutsos T., Isbister G.K. (2023). Snakebite-associated thrombotic microangiopathy: a spotlight on pharmaceutical interventions. Expet Rev. Clin. Pharmacol..

[bib26] Ostermann M., Zarbock A., Goldstein S., Kashani K., Macedo E., Murugan R., Bell M., Forni L., Guzzi L., Joannidis M., Kane-Gill S.L., Legrand M., Mehta R., Murray P.T., Pickkers P., Plebani M., Prowle J., Ricci Z., Rimmelé T., Rosner M., Shaw A.D., Kellum J.A., Ronco C. (2020). Recommendations on acute kidney injury biomarkers from the acute disease quality initiative consensus conference. JAMA Netw. Open.

[bib27] Pinho F.M.O., Yu L., Burdmann E.A. (2008). Kidney injury in Latin America. Semin. Nephrol..

[bib28] Rodrigues C.E., Endre Z.H. (2023). Definitions, phenotypes, and subphenotypes in acute kidney injury—moving towards precision medicine. Nephrology.

[bib29] Romejko K., Markowska M., Niemczyk S. (2023). The review of current knowledge on neutrophil gelatinase-associated lipocalin (NGAL). Int. J. Mol. Sci..

[bib30] Saharinen P., Eklund L., Alitalo K. (2008).

[bib31] Seifert S.A., Armitage J.O., Sanchez E.E. (2022). Snake envenomation. N. Engl. J. Med..

[bib32] Senthilkumaran S., Thirumalaikolundusubramanian P., Elangovan N. (2019). Neutrophil gelatinase-associated lipocalin as an early diagnostic biomarker of acute kidney injury in snake bite. J. Emergencies, Trauma, Shock.

[bib33] Sgrignolli L.R., Mendes G.E.F., Carlos C.P., Burdmann E.A. (2011). Acute kidney injury caused by Bothrops snake venom. Nephron Clin. Pract..

[bib34] Stanski N.L., Rodrigues C.E., Strader M., Murray P.T., Endre Z.H., Bagshaw S.M. (2023). Precision management of acute kidney injury in the intensive care unit : current state of the art. Intensive Care Med..

[bib35] Szumilas D., Owczarek A.J., Brzozowska A., Niemir Z.I., Olszanecka-glinianowicz M., Chudek J. (2024). The value of urinary NGAL , KIM-1 , and IL-18 measurements in the early detection of kidney injury in oncologic patients treated with cisplatin-based chemotherapy. Int. J. Mol. Sci..

[bib36] Thomazini C.M., Sachetto A.T.A., de Albuquerque C.Z., de Moura Mattaraia V.G., de Oliveira A.K., Serrano S.M. de T., Lebrun I., Barbaro K.C., Santoro M.L. (2021). Involvement of von Willebrand factor and botrocetin in the thrombocytopenia induced by Bothrops jararaca snake venom. PLoS Neglected Trop. Dis..

[bib37] Wedasingha S., Isbister G., Silva A. (2020). Bedside coagulation tests in diagnosing venom-induced consumption coagulopathy in snakebite. Toxins.

[bib38] Wedasingha S., Silva A., Fakes K., Siribaddana S., Isbister G.K. (2024). Utility of three serum biomarkers for early detection of systemic envenoming following viper bites in Sri Lanka. Ann. Emerg. Med..

[bib39] Wen Y., Xu L., Melchinger I., Thiessen-Philbrook H., Moledina D.G., Coca S.G., Hsu C., Go A.S., Liu K.D., Siew E.D., Ikizler T.A., Chinchilli V.M., Kaufman J.S., Kimmel P.L., Himmelfarb J., Cantley L.G., Parikh C.R. (2023). Longitudinal biomarkers and kidney disease progression after acute kidney injury. JCI Insight.

[bib40] Yoon S.-Y., Kim J.-S., Jeong K.-H., Kim S.-K. (2022). Acute kidney injury: biomarker-Guided Diagnosis and management. Medicina.

[bib41] Zhang P.L., Rothblum L.I., Han W.K., Blasick T.M., Potdar S., Bonventre J.V. (2008). Kidney injury molecule-1 expression in transplant biopsies is a sensitive measure of cell injury. Kidney Int..

[bib42] Zheng Z., Li C., Shao G., Li J., Xu K., Zhao Z., Zhang Z., Liu J., Wu H. (2021). Hippo-YAP/MCP-1 mediated tubular maladaptive repair promote inflammation in renal failed recovery after ischemic AKI. Cell Death Dis..

