# Supplementary material for: Pathophysiological role of endothelial biomarkers in Bothrops sp. venom-induced renal dysfunction and the therapeutic effect of antivenom
Source: Toxicon X. 2025 May 20;26:100226. doi: 10.1016/j.toxcx.2025.100226 (PMC12167772; doi:10.1016/j.toxcx.2025.100226)
Supplement: Multimedia component 1 [file mmc1.docx]

*Title:* Snake Antivenom and its Effects on Kidney and Endothelial Biomarkers in Human *Bothrops* Accident

Nicole Coelho Lopes (coelhonicole20@gmail.com)^a^, Gdayllon Cavalcante Meneses (gdayllon@ufc.br)^b^, Ranieri Sales de Souza Santos (ranisales2@gmail.com)^a^, Leticia Machado de Araújo (leticiamachado.ar@gmail.com)^b^, Bruna Viana Barroso Martins (bruna.vbarroso@gmail.com)^b^, Katarina Maria dos Reis Araújo (katarinamariareis@gmail.com)^b^, Valéria Holanda Nogueira de Aquino (mdvaleria@gmail.com)^b^, Igor Moreira de Almeida (igormdal@gmail.com)^a^, Sandra Mara Brasileiro Mota (sandramarabrasileiro@gmail.com)^d^, Geraldo Bezerra da Silva Junior (geraldobezerrajr@yahoo.com.br)^e^, Camila Eleuterio Rodrigues (c.eleuteriorodrigues@unsw.edu.au)^f,g^, Elizabeth De Francesco Daher (ef.daher@yahoo.com.br)^c^, Polianna Lemos Moura Moreira Albuquerque (polianna.albuquerque@cdu.edu.au)^h^, Alice Maria Costa Martins (martinsalice@gmail.com)^a,b^.

^a^ Pharmacology Post-Graduate Program, Federal University of Ceará, Fortaleza, Ceará, Brazil.

^b^ Department of Clinical and Toxicological Analysis Federal University of Fortaleza, Fortaleza, Ceará, Brazil;

^c^ Medical Sciences Post-Graduate Program, Federal University of Ceará, Ceará, Brazil

^d^ Toxicological Information and Assistance Center, Instituto Doutor Jose Frota Hospital, Fortaleza, Ceará, Brazil

^e^ University of Fortaleza, School of Medicine, Fortaleza, Ceará, Brazil

^f^ Hospital das Clínicas, University of São Paulo School of Medicine, São Paulo, Brazil

^g^ University of New South Wales, Sydney, Australia

^h^ Charles Darwin University, Darwin, Australia

**Address for correspondence:** Polianna Lemos Moura Moreira Albuquerque. Charles Darwin University, Darwin, Australia. Casuarina Campus, Blue 1.1.71, Ellengowan Drive, Post code 0909. E-mail: polianna.albuquerque@cdu.edu.au.

**Supplementary material**

**Figure S1.** Correlation between Ang-2 and kidney damage biomarkers at each timepoint. (a) Ang-2 vs uNGAL/uCr before antivenom treatment; (b) Ang-2 vs uNGAL/uCr 10h after antivenom treatment; (c) Ang-2 vs uNGAL/uCr 20h after antivenom treatment; (d) Ang-2 vs uKIM-1/uCr before antivenom treatment; (e) Ang-2 vs uKIM-1/uCr 10h after antivenom treatment; (f) Ang-2 vs uKIM-1/uCr 20h after antivenom treatment.


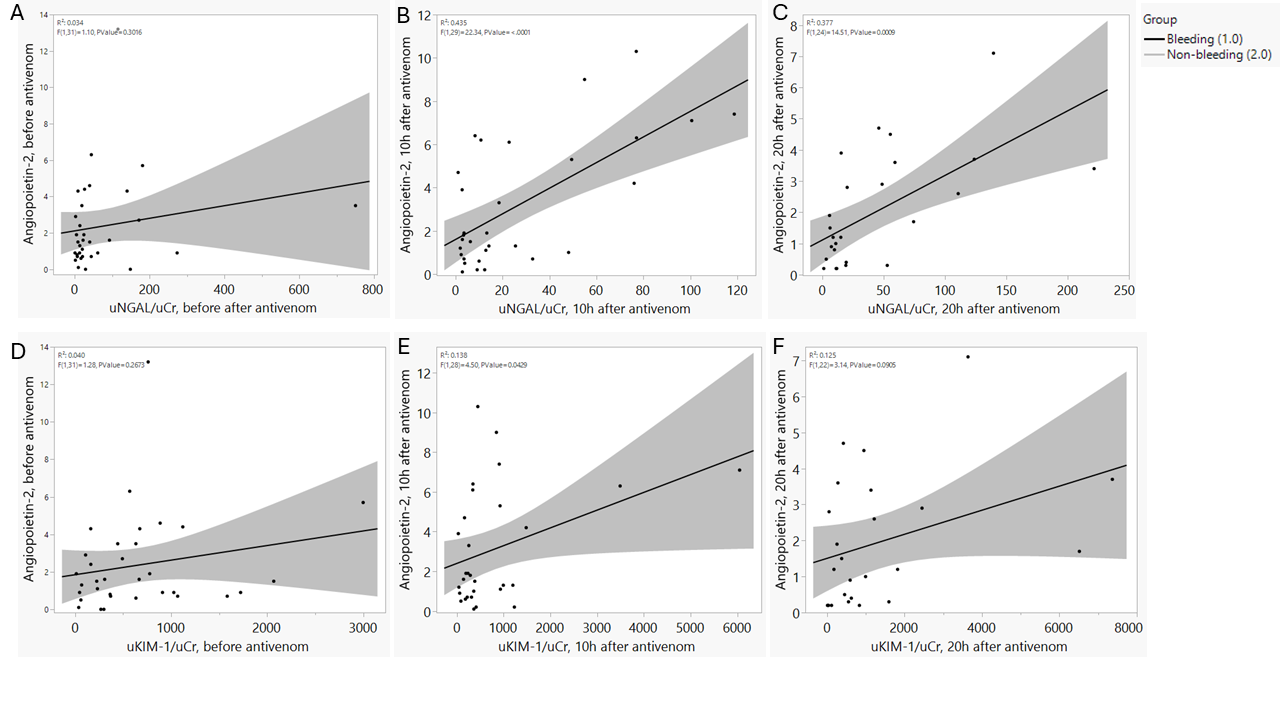


Ang-1: Angiopoietin-1. Ang-2: Angiopoietin-2. uCr: Urinary creatinine. uNGAL: Urinary neutrophil gelatinase-associated lipocalin. VCAM-1: vascular cell adhesion molecule-1.

**Figure S2.** Correlation between Ang-1 and kidney damage biomarkers at each timepoint. (a) Ang-1 vs uNGAL/uCr before antivenom treatment; (b) Ang-1 vs uNGAL/uCr 10h after antivenom treatment; (c) Ang-1 vs uNGAL/uCr 20h after antivenom treatment; (d) Ang-1 vs uKIM-1/uCr before antivenom treatment; (e) Ang-1 vs uKIM-1/uCr 10h after antivenom treatment; (f) Ang-1 vs uKIM-1/uCr 20h after antivenom treatment.


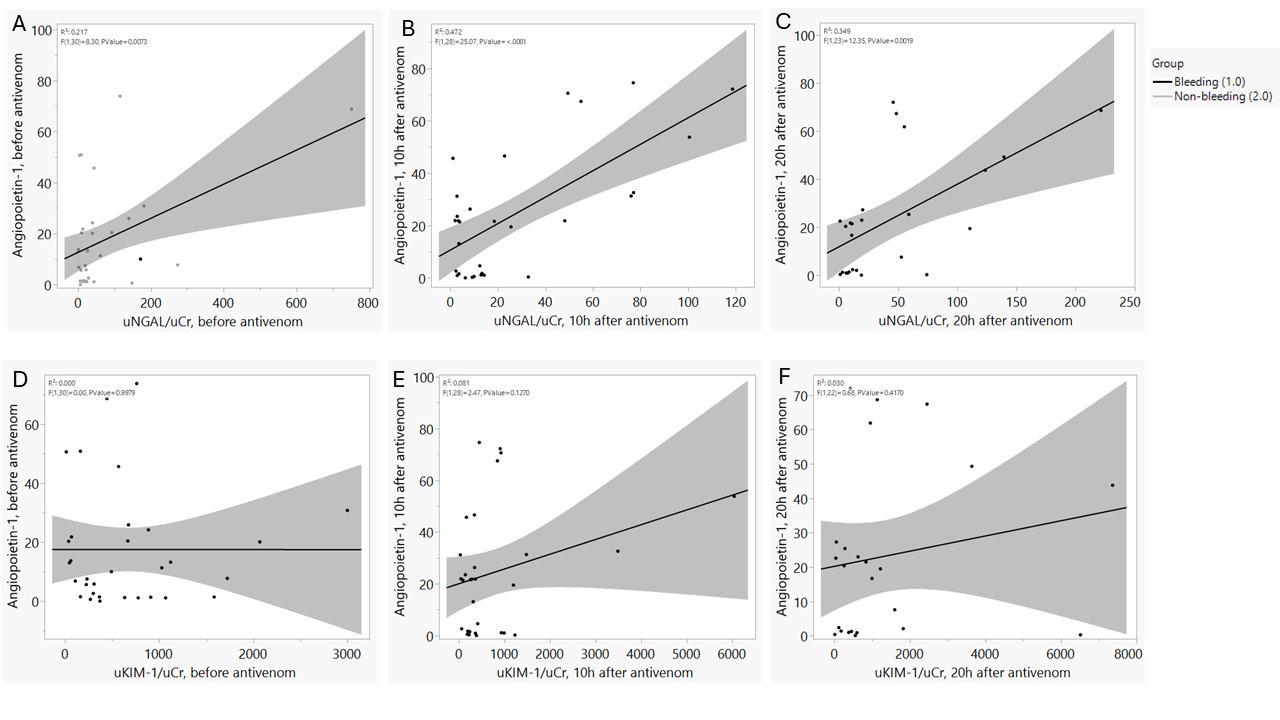


Ang-1: Angiopoietin-1. Ang-2: Angiopoietin-2. uCr: Urinary creatinine. uNGAL: Urinary neutrophil gelatinase-associated lipocalin. VCAM-1: vascular cell adhesion molecule-1.

**Figure S3.** Correlation between VCAM-1 and Ang-1 or Ang-2 by bleeding status at each timepoint. (a) VCAM-1 vs Ang-2 before antivenom treatment; (b) VCAM-1 vs Ang-2 at 10h after antivenom treatment; (c) VCAM-1 vs Ang-2 at 20h after antivenom treatment; (d) VCAM-1 vs Ang-1 before antivenom treatment; (e) VCAM-1 vs Ang-1 at 10h after antivenom treatment; (f) VCAM-1 vs Ang-1 at 20h after antivenom treatment.

**
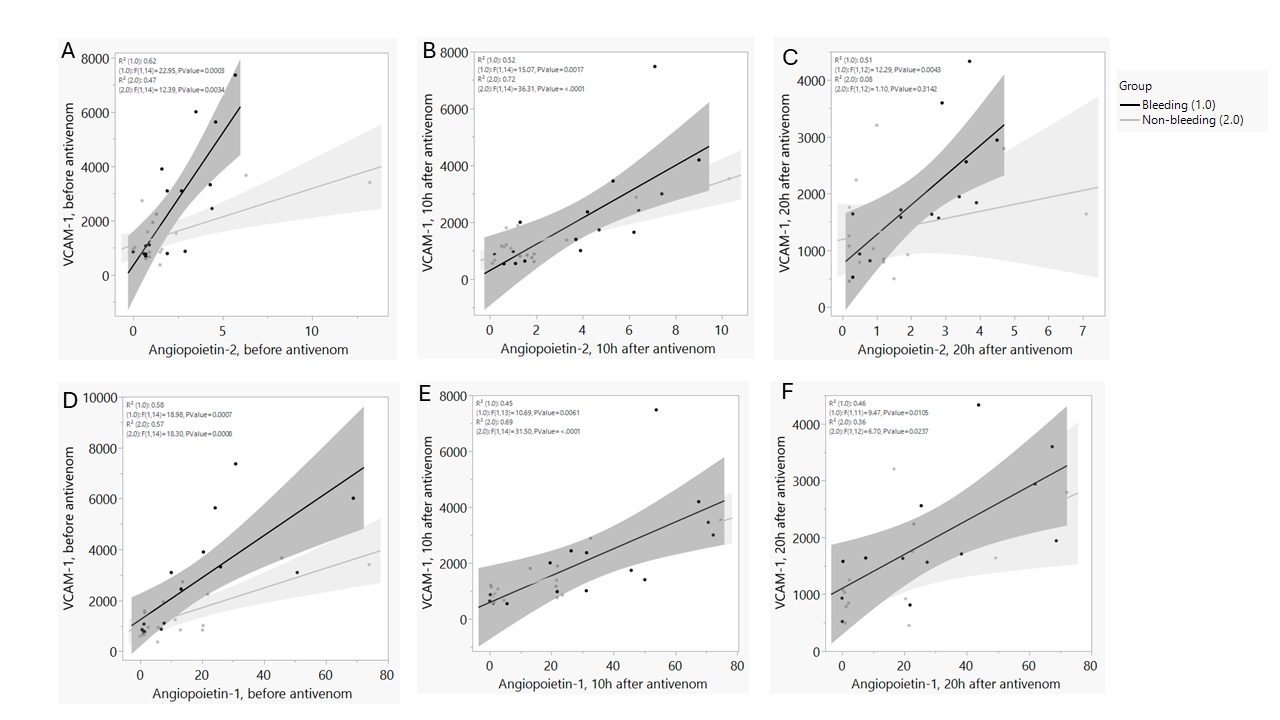
**

Ang-1: Angiopoietin-1. Ang-2: Angiopoietin-2. uCr: Urinary creatinine. uNGAL: Urinary neutrophil gelatinase-associated lipocalin. VCAM-1: vascular cell adhesion molecule-1.

**Tabela 3**

**Table S1.** Comparisons of uKIM-1/uCr levels before antivenom, 10h and 20h after antivenom extracted from linear mixed models in which fixed effects included bleeding, timepoints, the bleeding by time interactions and different endothelial markers measured before antivenom.

| Outcome: uKIM-1/uCr trajectory | Fixed effects | F ratio | p-value |
| --- | --- | --- | --- |
| Model 1 | Bleeding | 1.68 | 0.206 |
|  | Timepoints | 1.46 | 0.249 |
|  | Bleeding by time interaction | 0.14 | 0.870 |
|  | VCAM-1 level before antivenom | 1.39 | 0.249 |
| Model 2 | Bleeding | 3.32 | 0.079 |
|  | Timepoints | 1.54 | 0.229 |
|  | Bleeding by time interaction | 0.19 | 0.824 |
|  | Ang-1 level before antivenom | 0.003 | 0.953 |
| Model 3 | Bleeding | 3.51 | 0.071 |
|  | Timepoints | 1.52 | 0.234 |
|  | Bleeding by time interaction | 0.19 | 0.828 |
|  | Ang-2 level before antivenom | 1.80 | 0.191 |

Ang-1: Angiopoietin-1. Ang-2: Angiopoietin-2. uCr: Urinary creatinine. uKIM-1: Kidney Injury Molecule-1. VCAM-1: vascular cell adhesion molecule-1.
